# Exosomes containing miR‐451a is involved in the protective effect of cerebral ischemic preconditioning against cerebral ischemia and reperfusion injury

**DOI:** 10.1111/cns.13612

**Published:** 2021-02-03

**Authors:** He Li, Yin Luo, Peng Liu, Pei Liu, Weilong Hua, Yongxin Zhang, Lei Zhang, Zifu Li, Pengfei Xing, Yongwei Zhang, Bo Hong, Pengfei Yang, Jianmin Liu

**Affiliations:** ^1^ Stroke Center Changhai Hospital Shanghai China; ^2^ Department of neurosurgery Changhai Hospital Shanghai China; ^3^ Department of Biomedical Engineering School of Life Science and Technology Huazhong University of Science and Technology Wuhan China

**Keywords:** cerebral ischemic preconditioning, exosomes, ischemic and reperfusion injury, ischemic stroke

## Abstract

**Aim:**

To study the role of exosomes in the protective effect of cerebral ischemic preconditioning (cerebral‐IPC) against cerebral I/R injury.

**Method:**

Mouse models of cerebral‐IPC and MCAO/R were established as described previously, and their behavioral, pathological, and proteomic changes were analyzed. Neuro‐2a subjected to OGD/R were treated with exosomes isolated from the plasma of sham‐operated and cerebral‐IPC mice. The differentially expressed miRNAs between exosomes derived from sham‐operated (S‐exosomes) and preconditioned (IPC‐exosomes) mice were identified through miRNA array, and their targets were identified through database search. The control and OGD/R cells were treated with the IPC‐exosomes, miRNA mimic or target protein inhibitor, and their viability, oxidative, stress and apoptosis rates were measured. The activated pathways were identified by analyzing the levels of relevant proteins.

**Results:**

Cerebral‐IPC mitigated the cerebral injury following ischemia and reperfusion, and increased the number of plasma exosomes. IPC‐exosomes increased the survival of Neuro‐2a cells after OGD/R. The miR‐451a targeting Rac1 was upregulated in the IPC‐exosomes relative to S‐exosomes. The miR‐451a mimic and the Rac1 inhibitor NSC23766 reversed OGD/R‐mediated activation of Rac1 and its downstream pathways.

**Conclusion:**

Cerebral‐IPC ameliorated cerebral I/R injury by inducing the release of exosomes containing miR‐451a.

## Graphical abstract

Mechanisms underlying the protective effects of delayed CIPC against cerebral I/R injury.

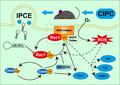

## 1 INTRODUCTION

Stroke is a common disorder of the central nervous system, with ischemic stroke accounting for 70–80% of all cases.^1^ Although mechanical thrombectomy can significantly increase the chances of successful reperfusion, the long‐term prognosis of stroke patients is poor due to ischemia and reperfusion (I/R) injury.^2^, ^3^, ^4^ Neuroprotective agents like edaravone and citicoline can alleviate the cerebral I/R injury in animal models but have not shown encouraging results in clinical trials.^5^, ^6^ In the recently concluded ESCAPE‐NA1 trial, the eicosapeptide nerinetide (NA‐1) did not significantly improve the clinical outcomes in acute ischemia stroke patients following thrombectomy and alteplase.^7^ Therefore, other strategies have to be explored in order to augment the therapeutic effect of reperfusion.

Ischemic preconditioning (IPC) is induced by transient and repeated (or long‐term but moderate) ischemia and reperfusion, which increases the tolerance of the target tissue to long‐term ischemia and I/R injury.^8^, ^9^ Direct‐IPC is induced by repeated I/R of the target organ, and remote‐IPC by repeated I/R of distal organs such as the extremities.^10^ Although remote‐IPC is technically simple, its efficacy has not been conclusively demonstrated in clinical studies.^11^ In contrast, direct cerebral‐IPC might be induced during transient ischemic attack (TIA) and intracranial atherosclerosis (ICAS).^12^, ^13^ In fact, patients with a history of TIA or ICAS tend to have smaller infarct volumes, indicating an endogenous protective effect of cerebral‐IPC. Therefore, it is necessary to elucidate the mechanism of cerebral‐IPC in order to identify novel neuroprotective targets.

Exosomes are extracellular vesicles measuring 50–150 nm in diameter that transport various bioactive components and thus regulate multiple physiological and even pathological processes.^14^ Li et al. found that remote‐IPC induces secretion of exosomes containing HIF‐1α, which protects against permanent distal middle cerebral artery occlusion.^15^ However, the neuroprotective effect of remote‐IPC and remote‐IPC‐derived exosomes does not extend to large vascular occlusion and I/R injury. Furthermore, the protective component in remote‐IPC‐derived exosomes is not equivalent to that in cerebral‐IPC‐derived exosomes.

In this study, we determined the efficacy of cerebral‐IPC and exosomes derived from cerebral‐IPC mice (IPC‐exosomes) in a mouse model of middle cerebral artery occlusion and reperfusion (MCAO/R), as well as a cellular model of oxygen‐glucose deprivation and restoration (OGD/R). To elucidate the underlying mechanisms, the miRNAs differentially expressed in the IPC‐exosomes were screened by miRNA array and confirmed by miR mimics. The target protein of the candidate miRNA was then confirmed, and the downstream pathways were identified.

## 2 MATERIALS AND METHODS

### 2.1 Establishment of MCAO/R and cerebral‐IPC models

Specific pathogen‐free (SPF) C57BL/6 male mice (8–10 weeks old and weighing 25–30 g) were purchased from Beijing Vital River Lab Animal Technology Co. Ltd., and housed in the SPF laboratory of Changhai Hospital Animals at 22°C and 12‐h day‐night cycle with ad libitum access to water and food. Seventy mice were divided into the sham‐operated, cerebral‐IPC, middle cerebral artery occlusion and reperfusion (MCAO/R), and cerebral‐IPC+MCAO/R groups. Cerebral‐IPC was induced by bilateral common carotid artery occlusion as previously described.^16^ Two‐hour MCAO/R was established 24 h after cerebral‐IPC using the suture‐occlusion method as previously described.^17^ Twenty‐four hours after the final operation, the neurological deficit score (NDS) of the mice was evaluated, and their plasma and brains were harvested for various assays (AppendixS 1).

### 2.2 Infarction volume measurement

The infarcted region in the brain was evaluated by TTC (2,3,5‐Triphenyltetrazolium chloride) staining. Briefly, freshly resected brains were sliced into 2 mm‐thick sections along the coronal axis and incubated in 2% TTC at 37°C. The vital region appeared red and the infarcted region remained white.

### 2.3 Measurement of neurological deficit score

The NDS was measured as previously described on a scale of 0–5: 0 ‐ no apparent deficiency, 1 ‐ flexion of contralateral forelimb, 2 ‐ rotated to the opposite side, 3 ‐ falling to the opposite side, 4 ‐ no spontaneous walk, and 5 ‐ dead.

### 2.4 Histological assessment

Brain tissues were fixed in 4% paraformaldehyde for 24 hours, dehydrated, embedded in paraffin, and sliced into 4‐μm‐thick sections. Hematoxylin and eosin (HE) staining, Nissl staining, and TUNEL staining were performed using specific kits from Servicebio Technology Co. Ltd. (Wuhan, China) as per the manufacturer's instructions.

### 2.5 Immunofluorescent staining

The paraffin‐embedded brain sections were probed with Iba‐1 (CST, 17198S) and NeuN (CST, 24307S) antibodies using an immunofluorescence kit (Servicebio Technology Co. Ltd.). The Iba‐1+, NeuN+, and DAPI+cells were, respectively, detected using 520 nm, 470 nm, and 364 nm excitation laser.

### 2.6 ROS (Reactive Oxidative Species) detection

Fresh brain tissues were embedded in optimal cutting temperature compound (OCT) and cut into 8–10 μm‐thick slices. The sections were stained using a fluorescent ROS probe using a specific kit from Servicebio Technology Co. Ltd. The ROS+and DAPI+cells were, respectively, detected using 520 nm and 364 nm excitation lasers.

### 2.7 Western blotting

Proteins were extracted from the cerebral cortex or cultured cells using the Membrane and Cytosol Protein Extraction Kit (Beyotime, China) and measured with the BCA Protein Assay Kit (Beyotime, China). Fifty micrograms protein per sample was separated through 10% ExpressPlus™ Gels (GenScript, the USA) and then transferred to Polyvinylidene fluoride (PVDF) membranes. After blocking with 5% skim milk in buffer, the membranes were incubated overnight at 4°C with the following antibodies: Caspase‐1 (89332, CST), Cleaved Caspase‐3 (ab214430, Abcam), COX‐2 (12282, CST), p‐JNK (ab124956, Abcam), JNK (ab179461, Abcam), p‐P38 (4511, CST), P38 (8690, CST), BAX (ab32504, Abcam), Bcl‐2 (ab182858, Abcam), MMP‐2 (87809S, CST), MMP‐9 (ab228402, Abcam), Catalase (ab76110, Abcam), Cu/Zn SOD (ab13498, Abcam), β‐actin (4970, CST), CD63 (EXOAB‐CD63A‐1, SBI), CD81 (EXOAB‐CD81A‐1, SBI), CD9 (EXOAB‐CD9A‐1, SBI), Rac1 (8631, CST), Cdc42 (8747, CST), PAK‐1(ab223849, Abcam) and p‐PAK1 (ab75599, Abcam). The membranes were washed and incubated with the secondary antibodies for 2 hours, and the positive bands were detected with a chromogenic developing agent.

### 2.8 MDA measurement

MDA concentration in the protein extracts of the brain or cultured cells was measured using the Lipid Peroxidation MDA Assay Kit (Beyotime, China) according to the manufacturer's instructions. The content of MDA was calculated per microgram protein (nmol/μg protein).

### 2.9 Isolation and characterization of plasma exosomes

Blood samples of the sham‐operated and cerebral‐IPC mice were collected for isolating exosomes. The mice were anesthetized again, and blood was drawn from the apex cordis and transferred into EDTA‐K2 tubes. After centrifuging at 2000 × g for 20 minutes at 4°C, the plasma layer was transferred into nuclease‐free tubes and centrifuged at 10,000 × g for 20 minutes at room temperature to remove debris. The supernatants were carefully removed and the exosomes were recovered from the pellets using the Total Exosome Isolation Kit (Invitrogen, ThermoFisher Scientific) as per the manufacturer's instructions. The exosomes were characterized as previously described.^18^ Briefly, the purified exosomes were re‐suspended in 500 μl PBS, and the total protein content was first measured using the BCA Protein Assay Kit (Beyotime Biotechnology). Nanoparticle tracking analysis (NTA) was performed to measure the size and concentration of exosomes as per standard protocols. Western blotting was performed as described above using antibodies against exosomal biomarkers including CD63, CD81, and CD9. Finally, the morphology of the exosomes was examined by electron microscopy.

### 2.10 Measurement of exosomal miRNAs

Exosomal miRNAs were extracted using the miRcute miRNA Isolation Kit (TianGen Biotech Co. Ltd.). The expression profiles of miRNAs in the different groups were compared by oligonucleotide microarray as described in previous studies and visualized using heatmaps and volcano plots.^19^ The results of high‐throughout sequencing were confirmed by quantitative real‐time PCR (qRT‐PCR). The primer sequences are listed in AppendixS 1.

### 2.11 Cell culture and treatment

Mouse neuroblastoma Neuro‐2a cells (N2a) were purchased from Shanghai Zhong Qiao Xin Zhou Biotechnology Co. Ltd. and cultured in MEM medium (ZQ‐300, Zhong Qiao Xin Zhou) supplemented with 10% fetal bovine serum (FBS, Zhong Qiao Xin Zhou) and 1% penicillin‐streptomycin (0513, ScienCell) under 5% CO_2_. The OGD/R model was established as previously described.^20^ The control and OGD/R cells were seeded in 96‐, 12‐, and 6‐well plates at the respective densities of 5 × 10^4^, 5 × 10^5^, and 1 × 10^6^ cells/well according to the experiment and incubated with the plasma exosomes derived from sham‐operated (S‐exosomes) and cerebral‐IPC (IPC‐exosomes) mice at ~1 × 10^11^ particles/ml. In addition, the N2a cells were also treated with 100 μM NSC23766 (HY‐15723A, MedChemExpress). The cellular grouping was recorded in AppendixS 1.

### 2.12 Cellular viability assay

Cell Counting Kit‐8 (CCK‐8; HY‐K0301, MedChemExpress) was used to measure the viability of the differentially treated cells according to the manufacturer's instructions. The absorbance of each group relative to the control was calculated.

### 2.13 Exosome uptake

Exosomes were labeled using the ExoGlow™‐Protein EV Labeling Kit (Red) (EXOGP100A‐1, System Bioscience) according to the manufacturer's instructions. The N2a cells cultured on sterile coverslips were incubated with the labeled exosomes for 24 hours and fixed with paraformaldehyde. Immunofluorescent staining was performed using NeuN antibody (AF1072, Beyotime) according to the instructions, and the stained cells were mounted with a drop of Antifade Mounting Medium containing DAPI (P0131, Beyotime). The internalized exosomes, N2a cells, and nucleus were, respectively, detected using 573 nm, 511 nm, and 364 nm excitation lasers.

### 2.14 Quantitative Real‐time PCR

The miRNA and mRNA levels in exosomes or cells were measured by qRT‐PCR. Total RNA was extracted using Trizol (Invitrogen, Thermo Fisher Scientific) according to the manufacturer's instructions, and the miRNA extraction protocol has been described above. The RNA samples were quantified at OD_260/280_, and reverse transcribed using the PrimeScriptTM RT reagent Kit (RR037A, TAKARA). QRT‐PCR was performed on LightCycler 96 (Roche Group) using SYBR® Premix Ex Taq™ II (Tli RNaseH Plus) (RR420A, TAKARA), and the fold change in expression levels was calculated by the 2^−△△Ct^ method. The primer sequences are listed in AppendixS 1.

### 2.15 MicroRNA mimic and transfection

The N2a cells were transfected with the miR‐451a mimic (5‘‐AAACCGUUACCAUUACUGAGUU‐3’). Briefly, miR‐451a mimic or negative control was incubated with Lipofectamine 3000 (Thermo Fisher Scientific), and the mixture was then added to the cultured cells. OGD/R was induced at least 24 hours after transfection.

### 2.16 TUNEL staining

TUNEL staining was performed by One Step TUNEL Apoptosis Assay Kit (C1086, Beyotime) according to the manufacturer's instructions. TUNEL+and DAPI+cells were detected using 470 nm and 364 nm excitation lasers, respectively.

### 2.17 ROS measurement

ROS levels were measured using the ROS Assay Kit (S0033, Beyotime) according to the manufacturer's instructions. The fluorescence intensity was measured at emission wavelength 535 nm and excitation wavelength 485 nm, and calculated relative to that of the control group.

### 2.18 GTP‐bound GTPase pull‐down assay

GTP‐bound GTPase pull‐down assay was used to determine the activation of Rac1 and Cdc42 as per the kit directions (8815 and 8819, Cell Signaling Technology). Activated Rac1 or Cdc42 were then eluted with sodium dodecyl sulfate and confirmed by Western blotting.

### 2.19 Statistical analysis

All statistical analysis was performed using GraphPad Prism 7.04. The normality of the data was verified by Shapiro–Wilk test. Two‐tailed t test was used to compare two groups, and one‐way ANOVA was used for multiple groups. *P* < 0.05 was considered statistically significant.

## 3 RESULTS

### 3.1 Cerebral‐IPC reduced cerebral infarction and ameliorated neurological dysfunction

**Figure 1:**
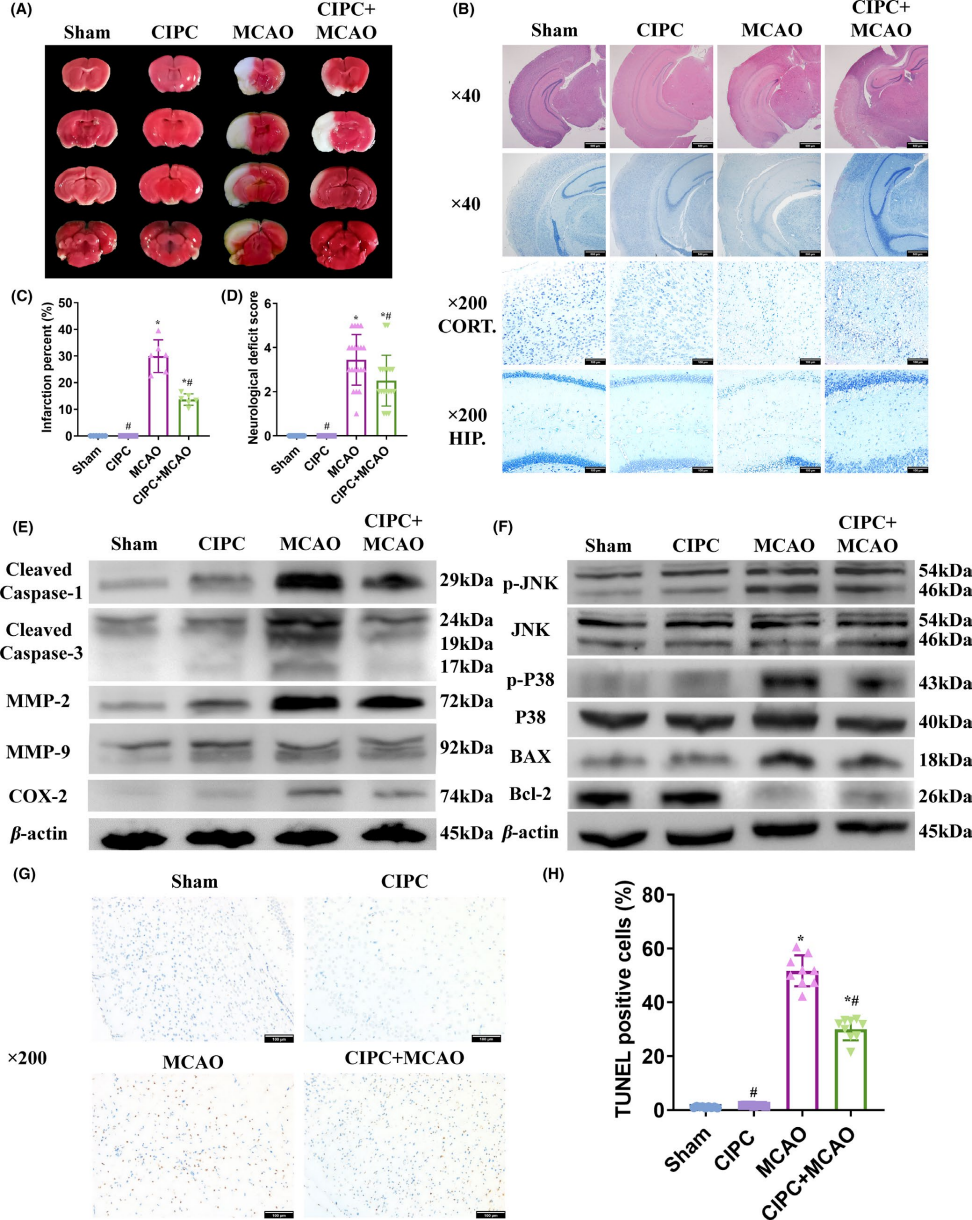
Cerebral‐IPC protects against MCAO/R injury. (A, C) Representative images of TTC staining showing infarcted regions in the brains in the differentially treated mice. (B) Representative images of HE and Nissl staining showing tissue structure and neuronal density in the differentially treated mice. (D) NDS in the differentially treated mice. (E) Representative immunoblot showing expression levels of COX2, MMP‐2, MMP‐9, cleaved caspase‐1, and cleaved caspase‐3 in the indicated groups. F: Representative immunoblot showing expression levels of JNK. P‐JNK, p38, p‐p38, BAX, and Bcl‐2 in the indicated groups. (G, H) Representative images of TUNEL‐stained tissues indicating apoptotic cells. (* and # *P* < 0.05 compared to Sham group and MCAO group, respectively; CIPC: cerebral‐IPC; Scale bars: 500 μm for ×40 and 100 μm for ×200)

**Figure 2:**
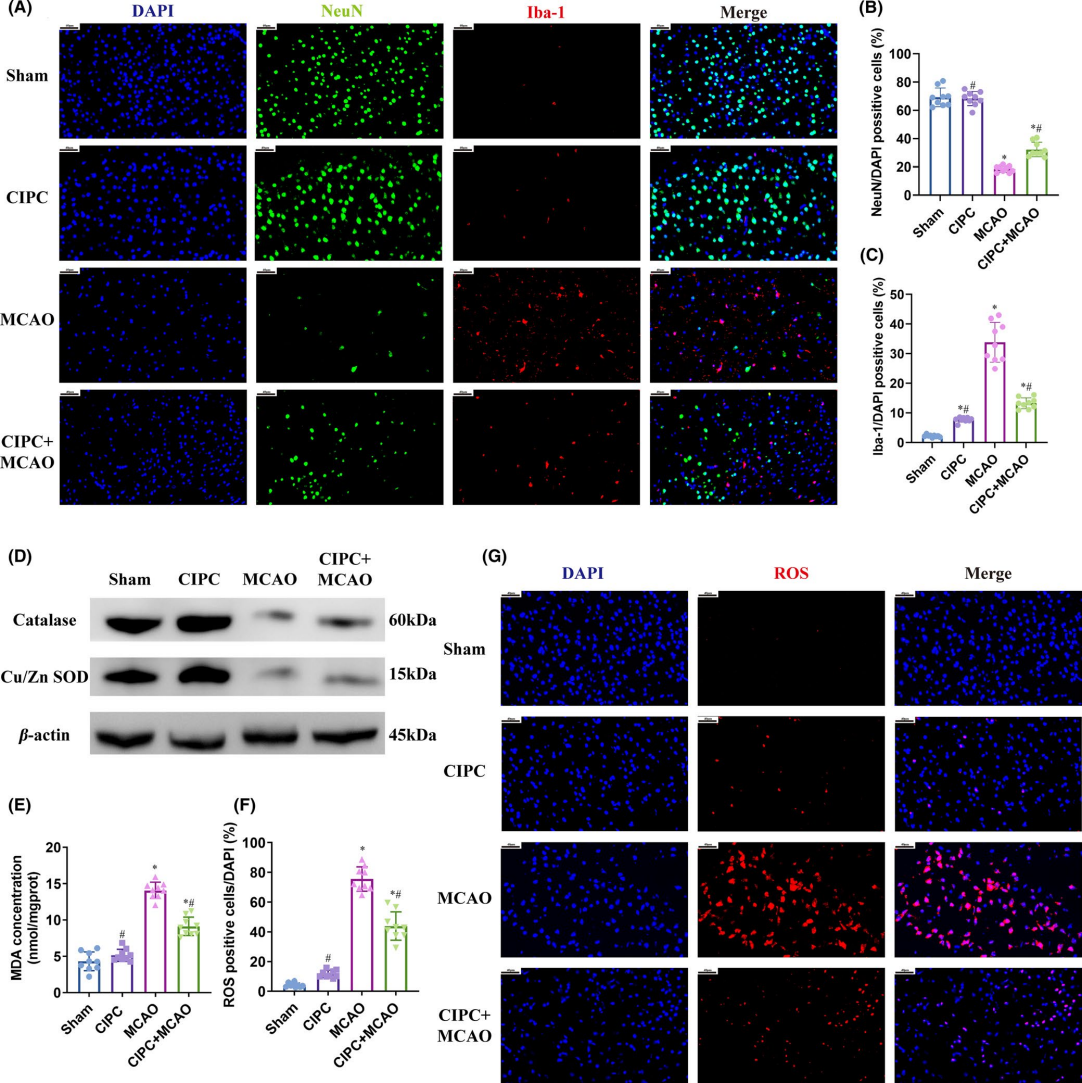
Cerebral‐IPC mitigates inflammation and oxidative stress after MCAO/R injury and increases neuronal survival. (A‐C) Representative immunofluorescence images showing NeuN+neurons and Iba‐1+ microglia in the indicated groups. D: Antioxidant enzyme levels in the indicated groups. E: MDA content in the indicated groups. F, G: The ratio of ROS positive cells in the indicated groups. (* and # *P* < 0.05 compared to Sham group and MCAO group, respectively; CIPC: cerebral‐IPC; Scale bars: 40 μm)

TTC staining showed that cerebral‐IPC did not induce significant infarction in the mouse brain, and in fact markedly decreased the infarcted region following MCAO/R (Figure 1A,C). Furthermore, MCAO/R caused significant tissue damage, neuronal death, and reduction in NeuN+cells, all of which were ameliorated by prior cerebral‐IPC (Figure 1B; Figure 2A,B). Furthermore, MCAO/R‐induced neurological dysfunction was also attenuated by cerebral‐IPC (Figure 1D). Taken together, cerebral‐IPC exerted a potent neuroprotective effect in mice with I/R injury.

### 3.2 Cerebral‐IPC ameliorated MCAO/R‐induced inflammation, apoptosis, and oxidative stress

MCAO/R significantly upregulated inflammatory and pro‐apoptotic markers including COX2, MMP‐2, MMP‐9, cleaved caspase‐1, and cleaved caspase‐3 (Figure 1E, AppendixS 1: Figure S1). In addition, the inflammatory and apoptotic pathways were activated by MCAO/R, as indicated by the increased levels of phosphorylated JNK and p38, and the respective induction and reduction in BAX and Bcl‐2. Cerebral‐IPC significantly attenuated the expression levels of the above factors (Figure 1F, AppendixS 1: Figure S2). Consistent with this, the percentage of TUNEL‐positive apoptotic cells and Iba‐1+ activated microglial cells were significantly lower in the cerebral‐IPC+MCAO compared with the MCAO group (Figure 1G,H; Figure 2A,C). Interestingly, cerebral‐IPC also induced mild inflammation and apoptosis, as indicated by the slight increase in the corresponding markers (Figure 1E) and the proportion of microglia compared to sham‐operated controls (Figure 2A,C). MDA, the final product of lipid peroxidation, was significantly elevated after MCAO/R, and attenuated by cerebral‐IPC (Figure 2E). MCAO/R‐mediated reduction in antioxidant enzymes including Cu/Zn SOD and catalase was also ameliorated by cerebral‐IPC (Figure 2D, AppendixS 1: Figure S3), which corresponded to a decrease in ROS levels (Figure 2F,G). Taken together, cerebral‐IPC elicited endogenous anti‐inflammatory, anti‐apoptotic and anti‐oxidative pathways in the injured brain, which was accompanied by slight tissue damage.

### 3.3 Cerebral‐IPC increased the exosomal load in plasma

**Figure 3:**
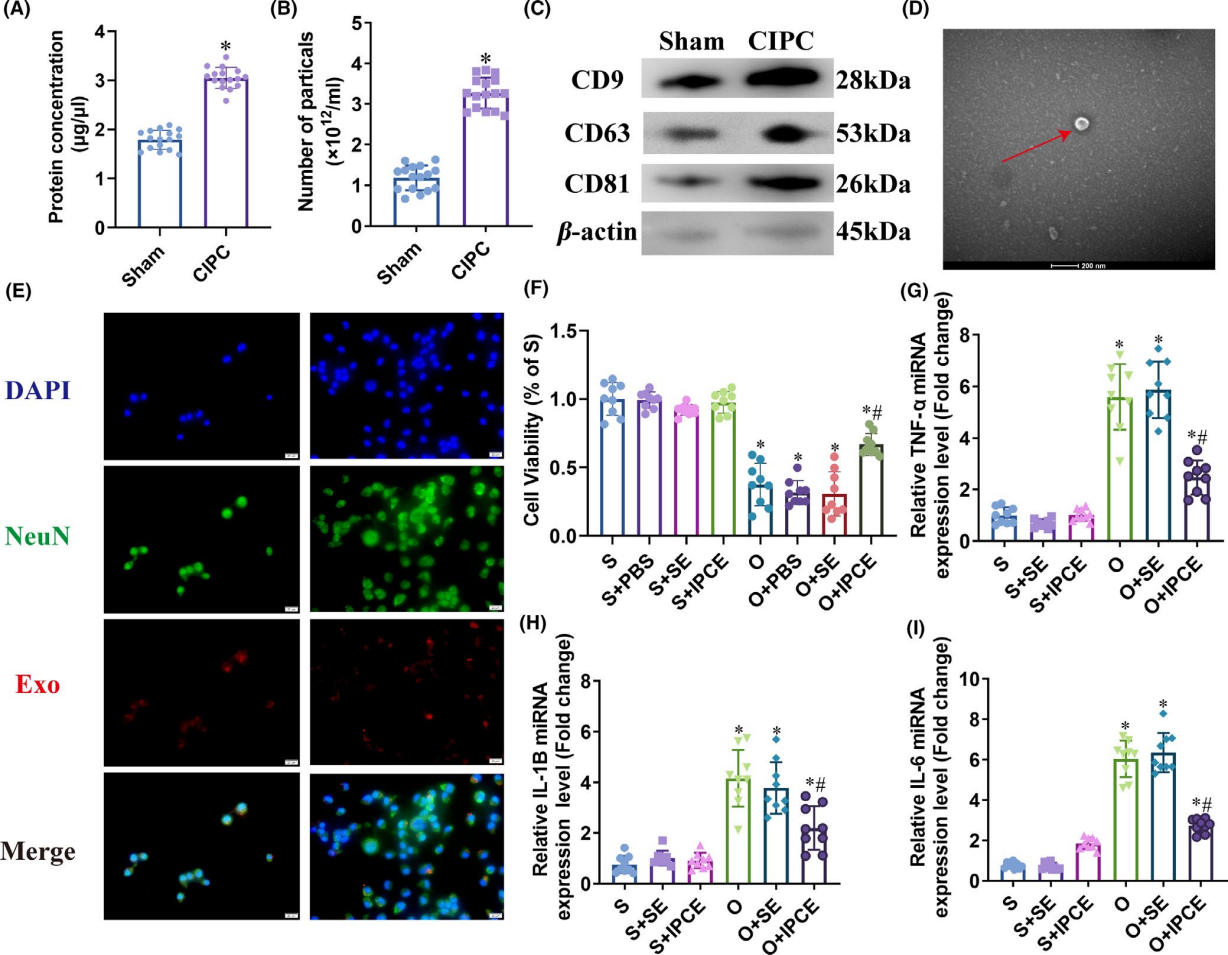
IPC‐exosomes promotes survival of N2a cells after OGD/R. (A) Exosomal protein concentration. (B) Number of exosomes in the plasma according to NTA. (C) Levels of exosomal biomarkers. (D) Representative electron micrographs showing the shape and size of exosomes. € Representative immunofluorescence images showing exosome internalization by N2a cells. (F) Percentage of viable cells in the indicated groups. (G‐I) Relative expression levels of pro‐inflammatory cytokine mRNAs in the indicated groups. (* and # *P* < 0.05 compared to S group and O group, respectively; SE:S‐exosomes, IPCE: IPC‐exosomes; Scale bars: 20 μm)

To determine the potential role of exosomes in the protective effect of cerebral‐IPC, we extracted exosomes from the plasma of the sham‐operated and cerebral‐IPC mice. The total amount of exosomal protein was significantly higher in the cerebral‐IPC versus the sham‐operated group (1.79 ± 0.20 μg/μL vs. 3.05 ± 0.21 μg/μL, *P* < 0.0001; Figure 3A). Furthermore, NTA showed that the number of particles with diameters ranging from 30 nm to 150 nm was also higher in the cerebral‐IPC mice compared to the sham‐operated controls (1.18 ± 0.31 × 10^12^ particles/ml vs. 3.27 ± 0.38 × 10^12^ particles/ml, *P* < 0.0001; Figure 3B). Finally, cerebral‐IPC increased the relative expression levels of the exosomal surface markers CD9, CD63, and CD81 (Figure 3C). The shape and size of the exosomes are shown in the electron micrographs in Figure 3D. To summarize, cerebral‐IPC elevated the number of exosomes in plasma, which likely mediate the protective effects of cerebral‐IPC against I/R injury.

### 3.4 Cerebral‐IPC‐derived exosomes (IPC‐exosomes) increased survival of N2a cells after OGD/R

**Figure 4:**
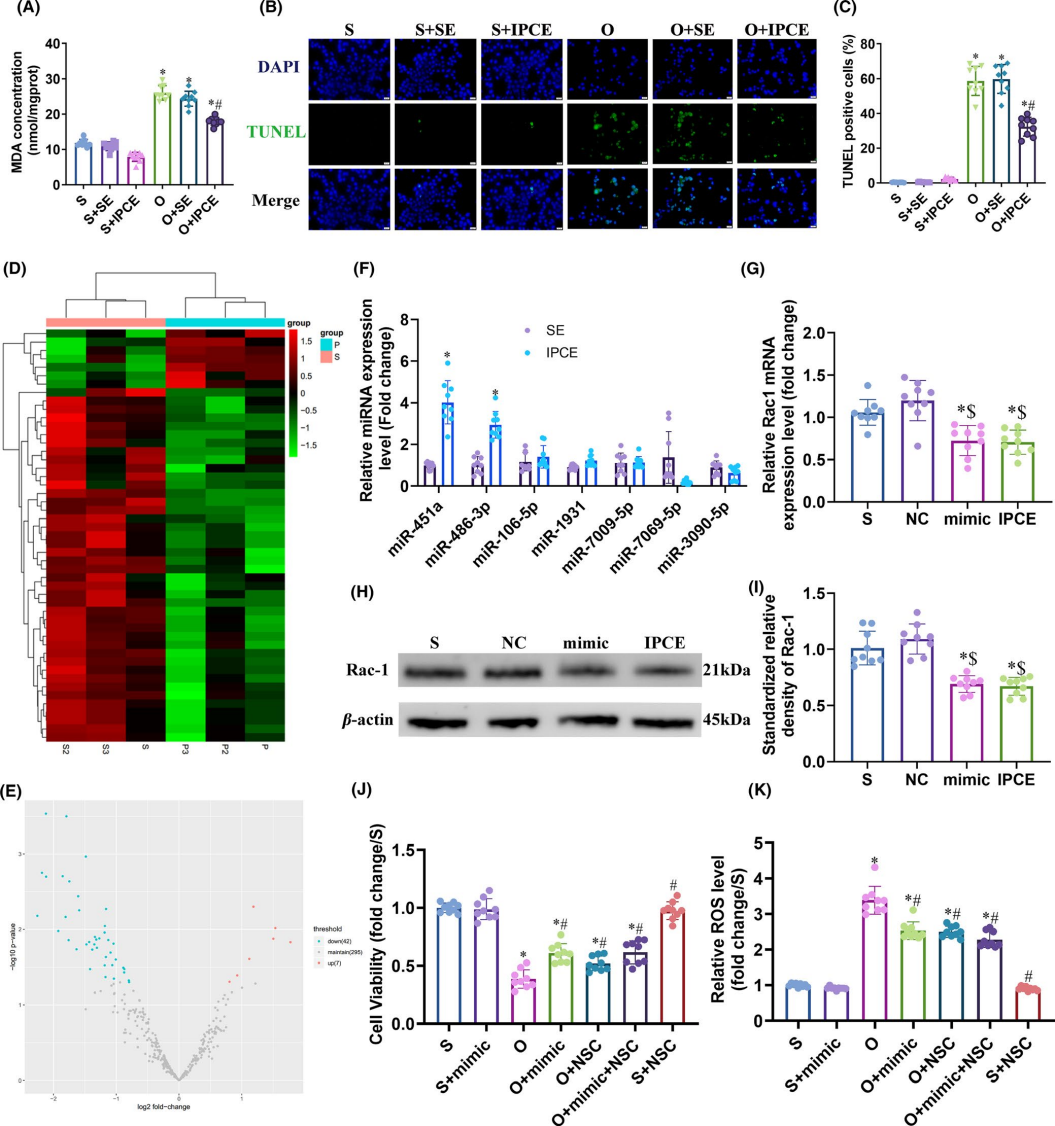
MiR‐451a is significantly upregulated in IPC‐exosomes and targets Rac1. (A) MDA content in the indicated groups. (B, C) Representative immunofluorescence images showing TUNEL‐positive apoptotic cells in the indicated groups. (D, E) Heat map and volcano plot showing differentially expressed miRNAs in the IPC‐exosomes relative to S‐exosomes. (F) Validation of upregulated miRNAs by qRT‐PCR. (G) Rac1 mRNA expression levels in the indicated groups. (H, I) Rac1 protein expression levels in the indicated groups. (J) Percentage of viable cells in the indicated groups. K: ROS levels in the indicated groups. (*, # and $ *P* < 0.05 compared to Sham/S, O and NC groups, respectively; NC: negative control; SE:S‐exosomes, IPCE: IPC‐exosomes, mimic: miR‐451a mimic, NSC: NSC23766)

An *in vitro* model of I/R injury was established by subjecting N2a cells to OGD/R, and the effects of the IPC‐exosomes were evaluated. As shown in Figure 3E, the N2a cells effectively internalized the fluorescent‐labeled exosomes. Furthermore, IPC‐exosomes increased the viability of cells after OGD/R stimulation compared to that of the untreated and S‐exosomes‐treated OGD/R cells (O+IPCE vs. O and O+SE: 0.69 ± 0.08 vs. 0.37 ± 0.16 and 0.31 ± 0.16, *P* < 0.0001 and *P* < 0.0001; Figure 3F). No significant difference was seen between the OGD and S‐exosomes +OGD groups. OGD/R treatment also increased the transcript levels of inflammatory factors including IL‐1b, IL‐6, and TNF‐α, which was partially attenuated by IPC‐exosomes (Figure 3G‐I). The MDA content was also significantly elevated after OGD/R treatment (11.86 ± 1.02 nmol/mg protein vs. 26.01 ± 2.04 nmol/mg protein, *P* < 0.0001) and reduced by IPC‐exosomes but not by S‐exosomes (26.01 ± 2.04 nmol/mg protein vs. 17.93 ± 1.13 nmol/mg protein, *P* < 0.0001; Figure 4A). TUNEL staining further showed that the proportion of apoptotic cells was significantly lower in the OGD +IPC‐exosomes group compared to the OGD group (32.32 ± 5.36% vs. 58.66 ± 8.34%, *P* < 0.0001; Figure 4B,C). Taken together, the IPC‐exosomes protected N2a cells from OGD/R‐induced injury by mitigating the latter's inflammatory, oxidative, and apoptotic effects.

### 3.5 MiR‐451a and miR‐486‐3p are upregulated in the IPC‐exosomes

To further dissect the protective mechanisms of IPC‐exosomes described so far, we analyzed the miRNA profiles of the S‐exosomes and IPC‐exosomes. As shown in Figure 4D,E, 49 miRNAs were differentially expressed in the IPC‐exosomes relative to S‐exosomes, of which 42 were downregulated and 7 were upregulated. The expression levels of the upregulated miRNAs, including miR‐106b‐5p, mmu‐miR‐451a, mmu‐miR‐1931, mmu‐miR‐3090‐5p, mmu‐miR‐486‐3p, mmu‐miR‐7009‐5p and mmu‐miR‐7069‐5p, were verified by qRT‐PCR. As shown in Figure 4F, only miR‐451a (Fold change: 4.07 ± 1.05, *P* < 0.0001) and miR‐486‐3p (Fold change: 2.87 ± 0.62, *P* < 0.0001) were significantly increased in the IPC‐exosomes. Since miR‐451a was 4.07‐fold upregulated in the IPC‐exosomes relative to S‐exosomes (*P* < 0.0001), we analyzed the biological relevance of this miRNA.

### 3.6 MiR‐451a targets Rac1 and its downstream pathways

We identified the putative targets of miR‐451a by screening the online databases (AppendixS 1). Rac1, Klhl5, AW549877, and Cep55 were identified in all databases, of which Rac1, a member of Ras superfamily, has established pro‐oxidative and pro‐inflammatory effects that may exacerbate the tissue damage caused by I/R injury. Furthermore, the downstream molecules of Rac1 include JNK, p38, MMP‐2, and MMP‐9, which were elevated after MCAO/R and reduced by cerebral‐IPC (Figure 1E,F). Therefore, we hypothesized that the high levels of miR‐451a in IPC‐exosomes protect against cerebral I/R injury by repressing the Rac1 expression. To confirm this hypothesis, we transfected the N2a cells with the miR‐451a mimic and found that Rac1 mRNA level was significantly reduced (1.06 ± 0.15 vs. 0.72 ± 0.17, *P* = 0.0026; Figure 4G). In addition, cells treated with IPC‐exosomes had lower Rac1 mRNA levels (1.06 ± 0.15 vs. 0.71 ± 0.14, *P* = 0.0014; Figure 4G), which was verified by the Western blotting as well (Figure 4H,I). Furthermore, the miR‐451a mimic promoted cellular survival (Figure 4J) and decreased ROS levels (Figure 4K) after OGD/R injury. NSC23766, a specific inhibitor of Rac1, also showed a similar protective effect against OGD/R injury (Figure 4J,K). These findings clearly indicated that miR‐451a exerts its protective effect by downregulating Rac1.

**Figure 5:**
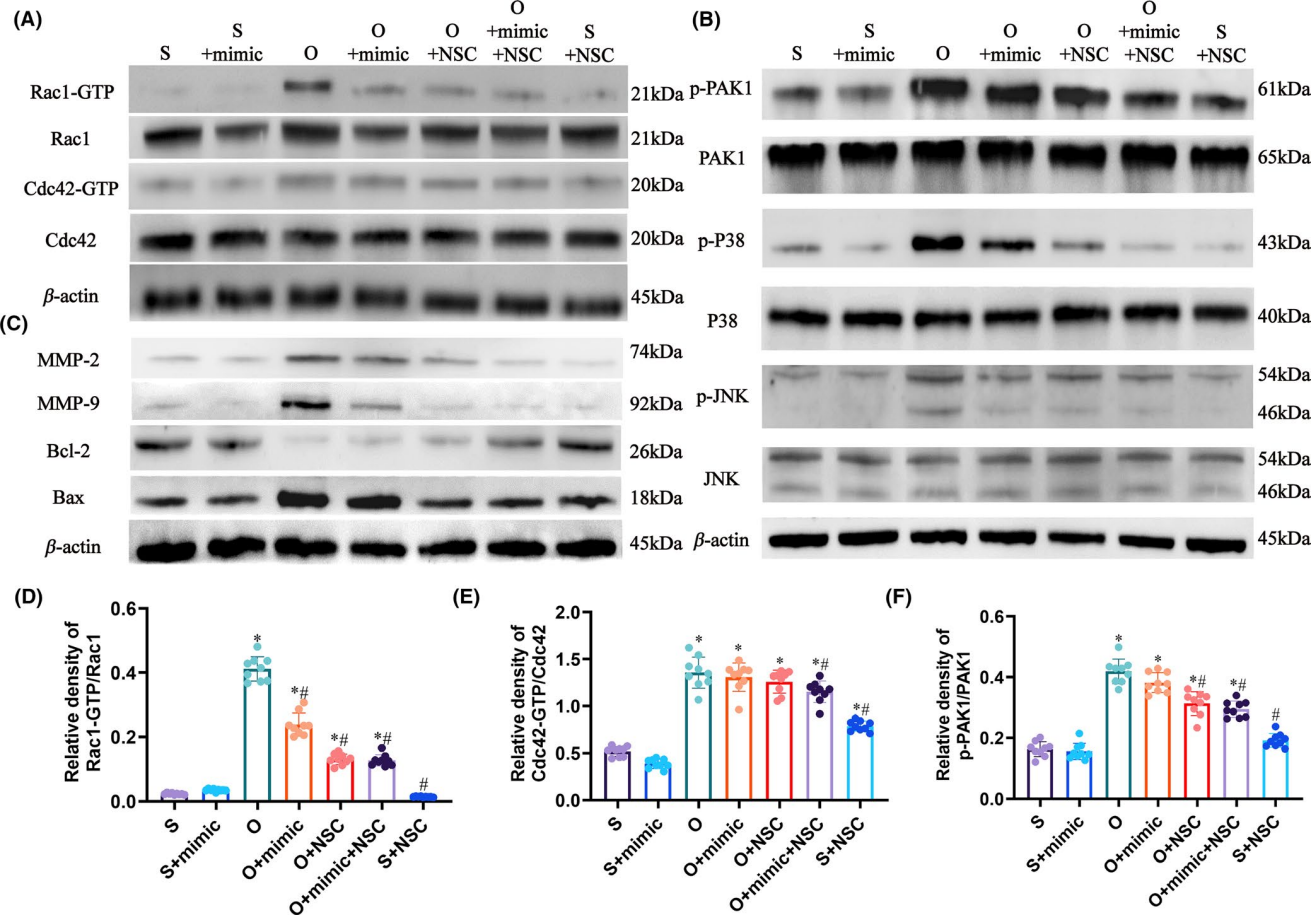
MiR‐451a suppresses the total expression and the activation of Rac1. (A) Representative immunoblot showing the expression levels of Rac1, Cdc42 and their activated forms in the indicated groups. (B, C) Representative immunoblot showing the expression levels of the downstream pathways of Rac1 in the indicated groups. (D) Rac1‐GTP/Total Rac1 ratio in the indicated groups. (E) Cdc42‐GTP/Total Cdc42 in the indicated groups. F: p‐PAK1/Total PAK1 ratio in the indicated groups. (* and # *P* < 0.05 compared to S and O groups, respectively; mimic: miR‐451a mimic, NSC: NSC23766)

**Figure 6:**
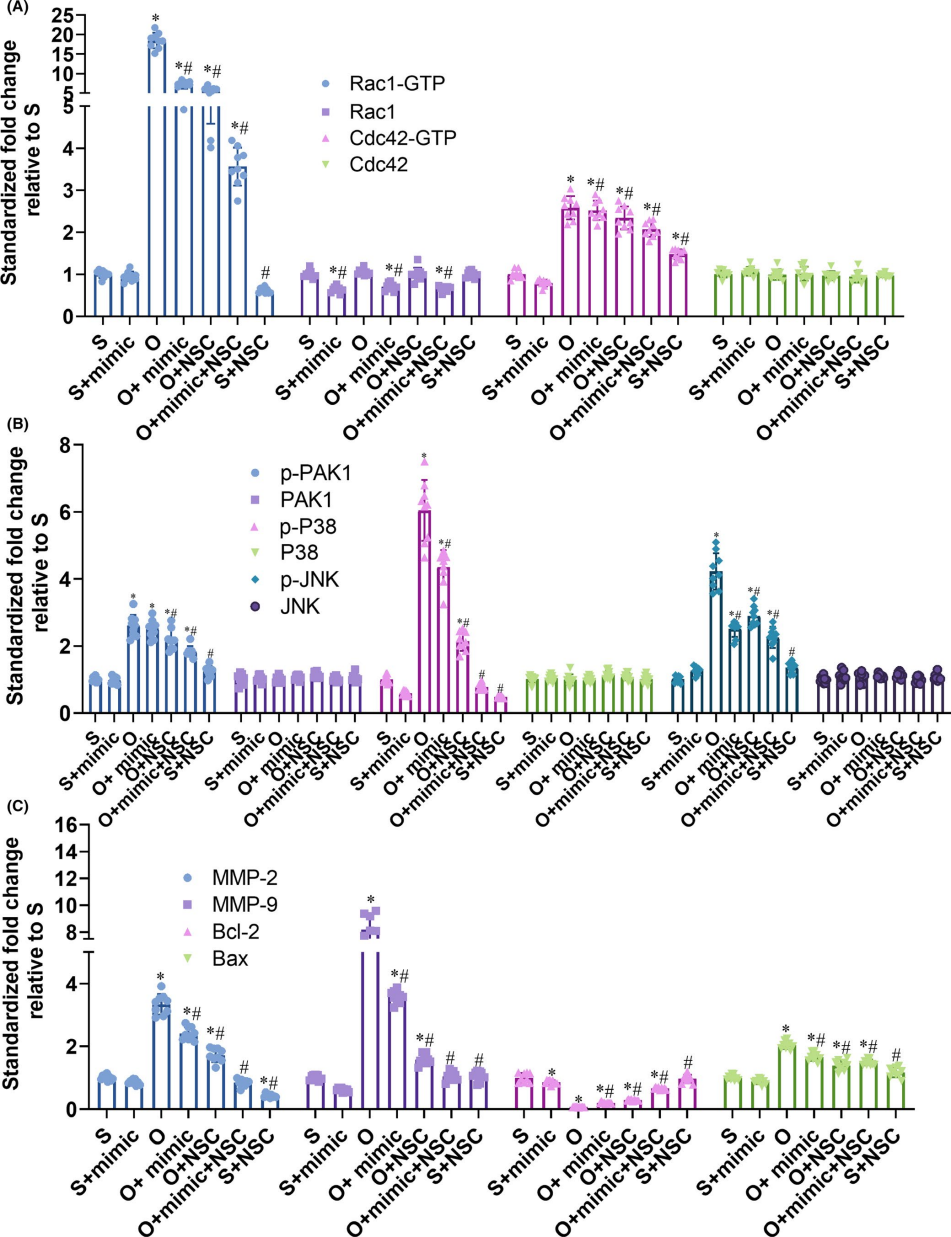
MiR‐451a suppresses the activation of downstream pathways of Rac1. (A‐C) The statistical analysis of the protein expression levels in the indicated groups according to the immunoblot. (* and # *P* < 0.05 compared to S and O groups, respectively; mimic: miR‐451a mimic, NSC: NSC23766)

Furthermore, the level of activated Rac1 (Rac1‐GTP) relative to Rac1 was significantly increased after OGD/R injury and reduced by miR‐451a mimic (0.41 ± 0.04 vs. 0.24 ± 0.04, *P* < 0.0001; Figure 5A,D). NSC23766 also reduced Rac1 activation without decreasing the total amount of Rac1 (0.53 ± 0.034 vs. 0.16 ± 0.029, *P* < 0.0001; Figure 5A,D, Figure 6A). Cdc42 is another member of Ras superfamily. Neither miR‐451a mimic nor NSC23766 alone affected the level of activated Cdc42 (Cdc42‐GTP) relative to Cdc42 after OGD/R injury, which indicated the specificity of miR‐451a and NSC23766 (Figure 5A,E, Figure 6A). PAK1 is a direct downstream molecule of Rac1 and Cdc42, and the relative expression level of p‐PAK1 to PAK1 was slightly reduced by miR‐451a mimic (0.42 ± 0.04 vs. 0.38 ± 0.03, *P* = 0.1658; Figure 5B,F). In contrast, NSC23766 showed a significant inhibitory effect on the relative expression of p‐PAK1 (0.42 ± 0.04 vs. 0.31 ± 0.03, *P* < 0.0001; Figure 5B,F, Figure 6B). Thus, miR‐451a‐mediated reduction in total Rac1 level differs from the inhibitory effect of NSC23766. However, the effects of miR‐451a mimic and NSC23766 were consistent on the JNK and P38 pathways, as well as on the expression levels of Bcl‐2, BAX, MMP‐2, and MMP‐9 (Figure 5B,C, Figure 6B,C). Taken together, miR‐451a protects against OGD/R injury by reducing total Rac1 expression and suppressing the Rac1‐mediated pathways.

## 4 DISCUSSION

We found that cerebral‐IPC protected mice against cerebral I/R injury, and the protective effects were mediated via the increased plasma load of IPC‐exosomes. The latter also exerted a similar protective effect on a cellular model of OGD/R injury. MiR‐451a was identified as one of the protective components in IPC‐exosomes that targets Rac1 and inhibits its downstream pathways (Graphic Abstract).

Several protective mechanisms of cerebral‐IPC have been demonstrated, such as reduction in cerebral metabolic rate, inhibition of excitotoxicity, oxidative stress and inflammation, and activation of neuroprotective or survival pathways.^21^, ^22^ In our study, cerebral‐IPC mitigated MCAO/R‐induced inflammation, apoptosis, and oxidative stress by inhibiting the JNK and P38 pathways. However, cerebral‐IPC by itself also triggered several inflammatory and apoptotic proteins and increased the proportion of active microglia, albeit to a significantly lesser extent compared to MCAO/R. Since cerebral‐IPC is induced by transient and/or repeated ischemic or anoxic insult, it may lead to brain damage on account of the high susceptibility of the brain to ischemia and anoxia. While the efficacy of remote‐IPC has been demonstrated as an alternative for cerebral‐IPC, it remains to be confirmed clinically.^11^ Direct cerebral‐IPC is still an indispensable method for studying cerebral endogenous protection. Thus, cerebral‐IPC is a double‐edged sword, and its underlying mechanisms need further study to augment the benefits and prevent the potential adverse effects.

Cerebral‐IPC significantly increased the number of plasma exosomes, which raised the possibility that these IPC‐exosomes mediated the neuroprotective effects after I/R injury. Exosomes are extracellular vesicles that transport proteins, miRNAs, lncRNAs, and circRNAs to remotely regulate physiological and pathological processes.^14^ Studies show that exosomes derived from endothelial progenitor cells, adipose‐derived stem cells, and multipotent mesenchymal stromal cells can manifest neuroprotective or neurotrophic effects by transporting protective miRNAs.^23^, ^24^, ^25^ Consistent with this, the plasma exosomes of sham‐operated and cerebral‐IPC mice in our study showed distinct miRNA profiles. Most miRNAs in the IPC‐exosomes were downregulated and only seven were upregulated. Based on previous findings that preconditioned cells or organs release protective factors, we hypothesized that the upregulated rather than the downregulated exosomal miRNAs are neuroprotective.^15^, ^26^, ^27^ Indeed, the neuroprotective effect of miR‐451a, the most significantly upregulated miRNA in IPC‐exosomes, was confirmed *in vitro*. MiR‐486‐3p, another upregulated miRNA in the IPC‐exosomes, is an established biomarker of acute coronary syndrome and should be studied further for its role in cerebral I/R injury.^28^ In addition, the downregulated miRNAs such as miR‐16–1, miR‐15a, and miR‐27 may also have functional significance in cerebral I/R injury. For instance, miR‐16–1 and miR‐15a promote inflammatory and apoptosis, and inhibition of miR‐27 enhances neurogenesis.^29^, ^30^, ^31^ Down‐regulation of these pathological miRNAs may therefore ameliorate the effects of cerebral I/R.

Rac1 was confirmed as the target of miR‐451a through bioinformatics and molecular analyses. Rac1, or Rac family small GTPase 1, is a GTP‐binding protein regulating cell growth and cytoskeletal function.^32^ Both protective and detrimental effects of Rac1 have been reported in the neurological system. Several studies have shown that Rac1 protects the brain‐blood barrier and exerts a regenerative effect on axons after cerebral ischemia.^33^, ^34^ On the other hand, inhibition of Rac1 lowers ROS production, which is a significant pathological factor underlying I/R injury.^35^, ^36^ Rac1‐GTP, the activated form of Rac1, is an essential component of NADPH oxidase and therefore crucial for ROS generation.^37^ Our findings supported a pathological role of Rac1 during OGD/R, which led to a significant increase in ROS levels and exacerbated tissue damage by upregulating MMPs, p‐P38, and p‐JNK. We hypothesize that cerebral‐IPC induces secretion of exosomes containing miR‐451a that represses Rac1 expression and its downstream pathways, eventually mitigating the pathological responses. Furthermore, the protective effects of miR‐451a mimic and Rac1 inhibitor were similar in the OGD/R model, further underscoring the role of Rac1 in I/R injury. The combination of miR‐451a mimic and Rac1 inhibitor inactivated the GTPase Cdc42, whereas either had no effect alone, indicating the selectivity of miR‐451a. PAK1 is a downstream molecule of Rac1 and Cdc42, which may involve the negative regulation of NADPH oxidase.^38^ Interestingly, the Rac1 inhibitor but not miR‐451a mimic significantly reduced the level of p‐PAK1 after OGD/R, which indicated that miR‐451a predominantly inhibits the total expression of Rac1 and the formation of NADPH oxidase rather than directly reducing the activation of Rac1 and its downstream pathways.

There are some limitations in this study that ought to be addressed. Our study evaluated the protective effect of cerebral‐IPC during the acute phase of MCAO/R and its long‐term efficacy remains to be determined. Secondly, although we established miR‐451a as a protective factor of IPC‐exosomes, the presence of other protective molecules cannot be ruled out. We did not perform *in vivo* experiments to assess the function of IPC‐exosomes due to the technical and ethical challenges related to harvesting large amounts of IPC‐exosomes. Our future studies will focus on evaluating the long‐term effect of cerebral‐IPC on the pathological and functional changes of after MCAO/R, and screening for the protective factors in IPC‐exosomes.^39^ In addition, identifying the cellular origin of IPC‐exosomes may allow *in vitro* production of neuroprotective exosomes. Cultured‐cell‐derived exosomes and nanoparticles containing bioactive components have significant clinical potential and warrant further investigation.

## 5 CONCLUSION

Cerebral‐IPC protected mice from MCAO/R injury through antioxidant, anti‐inflammatory, and anti‐apoptotic mechanisms. The IPC‐exosomes likewise promoted the survival of N2a cells after OGD/R. MiR‐451a was identified as a protective factor in the IPC‐exosomes, which repressed the total expression of Rac1 and inactivated its downstream pathological pathways.

## DISCLOSURE

All authors declare that they have no competing interests.

## AUTHOR'S CONTRIBUTIONS

He Li: wrote the manuscript and performed the animal modeling. Yin Luo: modified the manuscript and guided the experiment. Peng Liu and Pei Liu: performed histological experiment. Weilong Hua: performed proteomic experiment. Yongxin Zhang: performed microarray. Zifu Li: performed cellular experiments. Pengfei Xing: extracted the exosomes and RNAs. Yongwei Zhang: performed qRT‐PCR. Bo Hong: gave clinical advices and revised the manuscript. Pengfei Yang and Jianmin Liu: developed the research and designed the experiments.

## Supporting information

Appendix S1

## Data Availability

The data that support the findings of this study are available from the corresponding author upon reasonable request.

## References

[1] Virani SS , Alonso A , Benjamin EJ , et al. Heart Disease and Stroke Statistics‐2020 Update: A Report From the American Heart Association. Circulation. 2020;141(9):e139‐e596. 10.1161/CIR.0000000000000757 31992061

[2] Vidale S , Agostoni E . Endovascular Treatment of Ischemic Stroke: An Updated Meta‐Analysis of Efficacy and Safety. Vasc Endovascular Surg. 2017;51(4):215‐219. 28424039 10.1177/1538574417698905

[3] Pan J , Konstas AA , Bateman B , Ortolano GA , Pile‐Spellman J . Reperfusion injury following cerebral ischemia: pathophysiology, MR imaging, and potential therapies. Neuroradiology. 2007;49(2):93‐102. 17177065 10.1007/s00234-006-0183-z PMC1786189

[4] Schaller B , Graf R . Cerebral ischemia and reperfusion: the pathophysiologic concept as a basis for clinical therapy. J Cereb Blood Flow Metab. 2004;24(4):351‐371. 15087705 10.1097/00004647-200404000-00001

[5] Aoki J , Kimura K , Morita N , et al. YAMATO Study (Tissue‐Type Plasminogen Activator and Edaravone Combination Therapy). Stroke. 2017;48(3):712‐719. 28119434 10.1161/STROKEAHA.116.015042

[6] Dávalos A , Alvarez‐Sabín J , Castillo J , et al. Citicoline in the treatment of acute ischaemic stroke: an international, randomised, multicentre, placebo‐controlled study (ICTUS trial). Lancet. 2012;380(9839):349‐357. 22691567 10.1016/S0140-6736(12)60813-7

[7] Hill MD , Goyal M , Menon BK , et al. Efficacy and safety of nerinetide for the treatment of acute ischaemic stroke (ESCAPE‐NA1): a multicentre, double‐blind, randomised controlled trial. Lancet. 2020;395(10227):878‐887. 32087818 10.1016/S0140-6736(20)30258-0

[8] Sprick JD , Mallet RT , Przyklenk K , Rickards CA . Ischaemic and hypoxic conditioning: potential for protection of vital organs. Exp Physiol. 2019;104(3):278‐294. 30597638 10.1113/EP087122 PMC6397065

[9] Bhuiyan MI , Kim YJ . Mechanisms and prospects of ischemic tolerance induced by cerebral preconditioning. Int Neurourol J. 2010;14(4):203‐212. 21253330 10.5213/inj.2010.14.4.203 PMC3021810

[10] Vinciguerra A , Cuomo O , Cepparulo P , et al. Models and methods for conditioning the ischemic brain. J Neurosci Methods. 2018;310:63‐74. 30287283 10.1016/j.jneumeth.2018.09.029

[11] Zhao W , Zhang J , Sadowsky MG , Meng R , Ding Y . Remote ischaemic conditioning for preventing and treating ischaemic stroke. Cochrane Database Syst Rev. 2018;7:CD012503. 29974450 10.1002/14651858.CD012503.pub2 PMC6513257

[12] Wegener S , Gottschalk B , Jovanovic V , et al. Transient ischemic attacks before ischemic stroke: preconditioning the human brain? A multicenter magnetic resonance imaging study. Stroke. 2004;35(3):616‐621. 14963288 10.1161/01.STR.0000115767.17923.6A

[13] Yang W , Zhang Y , Li Z , et al. Differences in Safety and Efficacy of Endovascular Treatment for Acute Ischemic Stroke. Clin Neuroradiol. 2020. 10.1007/s00062-020-00899-x 32239261

[14] Dreyer F , Baur A . Biogenesis and Functions of Exosomes and Extracellular Vesicles. Methods Mol Biol. 2016;1448:201‐216. 27317183 10.1007/978-1-4939-3753-0_15

[15] Li Y , Ren C , Li H , et al. Role of exosomes induced by remote ischemic preconditioning in neuroprotection against cerebral ischemia. NeuroReport. 2019;30(12):834‐841. 31283710 10.1097/WNR.0000000000001280

[16] Tu XK , Yang WZ , Chen JP , et al. Repetitive ischemic preconditioning attenuates inflammatory reaction and brain damage after focal cerebral ischemia in rats: involvement of PI3K/Akt and ERK1/2 signaling pathway. J Mol Neurosci. 2015;55(4):912‐922. 25338292 10.1007/s12031-014-0446-9

[17] Chiang T , Messing RO , Chou WH . Mouse model of middle cerebral artery occlusion. J Vis Exp. 2011;48:10. 10.3791/2761 PMC3197421 21372780

[18] Xu B , Zhang Y , Du XF , et al. Neurons secrete miR‐132‐containing exosomes to regulate brain vascular integrity. Cell Res. 2017;27(7):882‐897. 28429770 10.1038/cr.2017.62 PMC5518987

[19] Carvalho BS , Irizarry RA . A framework for oligonucleotide microarray preprocessing. Bioinformatics. 2010;26(19):2363‐2367. 20688976 10.1093/bioinformatics/btq431 PMC2944196

[20] Li T , You H , Mo X , et al. GOLPH3 Mediated Golgi Stress Response in Modulating N2A Cell Death upon Oxygen‐Glucose Deprivation and Reoxygenation Injury. Mol Neurobiol. 2016;53(2):1377‐1385. 25633094 10.1007/s12035-014-9083-0

[21] De Rivero Vaccari JP , Bramlett HM , Perez‐Pinzon MA , Raval AP . Estrogen preconditioning: A promising strategy to reduce inflammation in the ischemic brain. Cond Med. 2019;2(3):106‐113. 32617523 PMC7331970

[22] Bhowmick S , Drew KL . Mechanisms of innate preconditioning towards ischemia/anoxia tolerance: Lessons from mammalian hibernators. Cond Med. 2019;2(3):134‐141. 32542230 PMC7295161

[23] Wang J , Chen S , Zhang W , Chen Y , Bihl JC . Exosomes from miRNA‐126‐modified endothelial progenitor cells alleviate brain injury and promote functional recovery after stroke. CNS Neurosci Ther. 2020;26(12):1255‐1265. 33009888 10.1111/cns.13455 PMC7702230

[24] Liu CY , Yin G , Sun YD , et al. Effect of exosomes from adipose‐derived stem cells on the apoptosis of Schwann cells in peripheral nerve injury. CNS Neurosci Ther. 2020;26(2):189‐196. 31278850 10.1111/cns.13187 PMC6978230

[25] Xin H , Liu Z , Buller B , et al. MiR‐17‐92 enriched exosomes derived from multipotent mesenchymal stromal cells enhance axon‐myelin remodeling and motor electrophysiological recovery after stroke. J Cereb Blood Flow Metab. 2020. 10.1177/0271678X20950489 PMC8054728 32811262

[26] Guitart K , Loers G , Buck F , Bork U , M. Schachner.R. Kleene . Improvement of neuronal cell survival by astrocyte‐derived exosomes under hypoxic and ischemic conditions depends on prion protein. Glia. 2016;64(6):896‐910. 26992135 10.1002/glia.22963

[27] Guo X , Qiu W , Liu Q , et al. Immunosuppressive effects of hypoxia‐induced glioma exosomes through myeloid‐derived suppressor cells via the miR‐10a/Rora and miR‐21/Pten Pathways. Oncogene. 2018;37(31):4239‐4259. 29713056 10.1038/s41388-018-0261-9

[28] Wei T , Folkersen L , Ehrenborg E , Gabrielsen A . MicroRNA 486–3P as a stability marker in acute coronary syndrome. Biosci Rep. 2016;36:3. 10.1042/BSR20160023 PMC5293558 27190129

[29] Kang C , Gao J , Kang M , Liu X , Wang YFuL . Sappanone A prevents hypoxia‐induced injury in PC‐12 cells by down‐regulation of miR‐15a. Int J Biol Macromol. 2019;123:35‐41. 30395900 10.1016/j.ijbiomac.2018.11.002

[30] Wang Z , Yuan Y , Zhang Z , Ding K . Inhibition of miRNA‐27b enhances neurogenesis via AMPK activation in a mouse ischemic stroke model. FEBS Open Bio. 2019;9(5):859‐869. 10.1002/2211-5463.12614 PMC6487723 30974042

[31] Yang X , Tang X , Sun P , et al. MicroRNA‐15a/16‐1 Antagomir Ameliorates Ischemic Brain Injury in Experimental Stroke. Stroke. 2017;48(7):1941‐1947. 28546328 10.1161/STROKEAHA.117.017284 PMC5516963

[32] Acevedo A , Gonzalez‐Billault C . Crosstalk between Rac1‐mediated actin regulation and ROS production. Free Radic Biol Med. 2018;116:101‐113. 29330095 10.1016/j.freeradbiomed.2018.01.008

[33] Sherchan P , Huang L , Akyol O , Reis C , Tang J , Zhang JH . Recombinant Slit2 Reduces Surgical Brain Injury Induced Blood Brain Barrier Disruption via Robo4 Dependent Rac1 Activation in a Rodent Model. Sci Rep. 2017;7(1):746. 28389649 10.1038/s41598-017-00827-z PMC5429690

[34] Liu L , Yuan H , Yi Y , et al. Ras‐Related C3 Botulinum Toxin Substrate 1 Promotes Axonal Regeneration after Stroke in Mice. Transl Stroke Res. 2018;9(5):506‐514. 29476448 10.1007/s12975-018-0611-5 PMC6598679

[35] Pan Y , Wang N , Xia P , Wang E , Guo Q , Ye Z . Ye, Inhibition of Rac1 ameliorates neuronal oxidative stress damage via reducing Bcl‐2/Rac1 complex formation in mitochondria through PI3K/Akt/mTOR pathway. Exp Neurol. 2018;300:149‐166. 29129468 10.1016/j.expneurol.2017.10.030

[36] Karabiyik C , Fernandes R , Figueiredo FR , et al. Neuronal Rho GTPase Rac1 elimination confers neuroprotection in a mouse model of permanent ischemic stroke. Brain Pathol. 2018;28(4):569‐580. 28960571 10.1111/bpa.12562 PMC8028553

[37] Shen J , Bai XY , Qin Y , et al. Interrupted reperfusion reduces the activation of NADPH oxidase after cerebral I/R injury. Free Radic Biol Med. 2011;50(12):1780‐1786. 21458562 10.1016/j.freeradbiomed.2011.03.028

[38] Desantiago J , Bare DJ , Xiao L , Ke Y , Solaro RJ , Banach K . Banach, p21‐Activated kinase1 (Pak1) is a negative regulator of NADPH‐oxidase 2 in ventricular myocytes. J Mol Cell Cardiol. 2014;67:77‐85. 24380729 10.1016/j.yjmcc.2013.12.017 PMC3930036

[39] Zhang J , Zhang W , Gao X , et al. Preconditioning with partial caloric restriction confers long‐term protection against grey and white matter injury after transient focal ischemia. J Cereb Blood Flow Metab. 2019;39(7):1394‐1409. 29972653 10.1177/0271678X18785480 PMC6668518

