# Supplementary material for: Exosomes containing miR‐451a is involved in the protective effect of cerebral ischemic preconditioning against cerebral ischemia and reperfusion injury
Source: CNS Neurosci Ther. 2021 Feb 3;27(5):564–76. doi: 10.1111/cns.13612 (PMC8025619; doi:10.1111/cns.13612)
Supplement: Supplementary file 1 — Appendix S1 [file CNS-27-564-s001.docx]

**Exosomes containing miR-451a is involved in the protective effect of cerebral ischemic preconditioning against cerebral ischemia and reperfusion injury**

**Supplementary** **materials**

**1.** **Supplementary methods**

**1.1 Animal grouping**


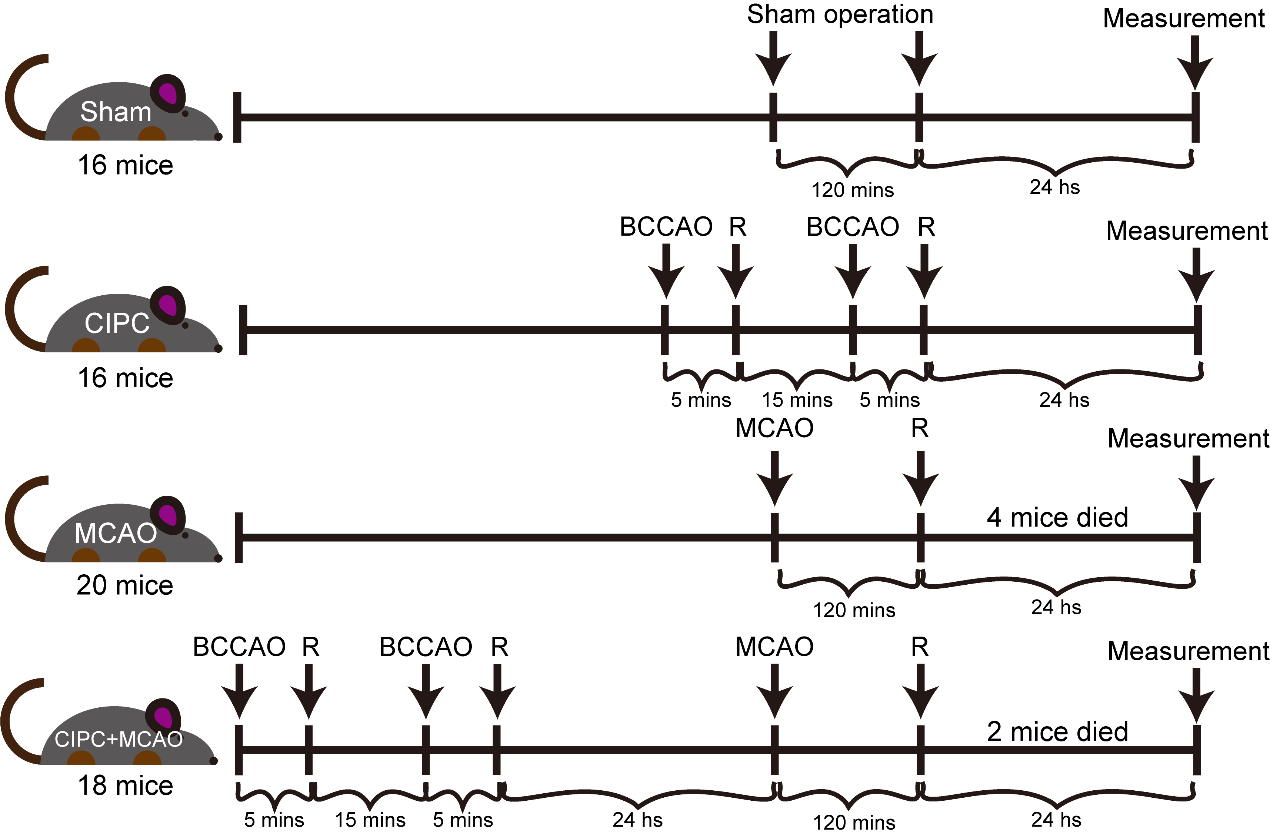


**Supplementary Figure.** Sixteen mice were assigned each to the Sham-operated and cerebral-IPC groups. Cerebral-IPC was induced by two cycles of BCCAO and reperfusion. MCAO was induced in 20 mice for 120 minutes, and 18 mice underwent cerebral-IPC prior to MCAO. Four mice died in the MCAO group, and two died in the cerebral-IPC+MCAO group. Twenty-four hours after the final operation, the NDS of the mice was measured, and plasma and brains were harvested for further experiments. (CIPC: cerebral-IPC; BCCAO: bilateral common carotid artery occlusion; MCAO: middle cerebral artery occlusion)

**1.2 Animal using**

The plasma of 32 mice from Sham (16) and MCAO groups (16) was harvested for exosome preparation. The mice brains from each group were harvested for further measurement. Six in 16 mice brains underwent TTC staining. Four in 16 mice brains were fixed and embedded in paraffin, preparing for HE, Nissl, TUNEL, and immunohistochemical staining. Three in 16 mice brains were embedded in OCT, preparing for ROS staining. Three in 16 mice brains were homogenized, preparing for ROS Western Blotting.

**1.3 Cellular grouping**

The cells were divided into eight groups when performing cellular viability, inflammation, and apoptosis assessment: (1)S: The cells were cultured in normal condition. (2)S+PBS: The cells received PBS treatment to exclude the effect of the solvent of exosomes. (This group is exclusively included in the cellular viability assessment.) (3)S+S-exosomes: The cells were co-cultured with exosomes derived from sham-operated mice (S-exosomes). (4)S+IPC-exosomes: The cells were co-cultured with exosomes derived from mice that underwent cerebral-IPC (IPC-exosomes). (4)O: The cells underwent OGD for 4 hours and restoration for 24 hours before assessment. (5)O+PBS: The cells received PBS treatment 24 hours before OGD/R. (This group is exclusively included in the cellular viability assessment.) (6)O+SE: The cells were co-cultured with S-exosomes for 24 hours before OGD/R. (7)O+IPC-exosomes: The cells were co-cultured with IPC-exosomes for 24 hours before OGD/R.

The cells were divided into four groups when confirming the target of miR-451a: (1)S: The cells were cultured in normal condition. (2)NC: The cells were transfected with negative controls. (3)mimic: The cells were transfected with miR-451a mimic. (4)IPC-exosomes: The cells were co-cultured with IPC-exosomes.

The cells were divided into seven groups when confirming the mechanism of miR-451a: (1)S: The cells were cultured in normal condition. (2)S+miR-451a mimic: The cells were co-cultured with miR-451a mimic for 24 hours. (3)O: The cells underwent OGD for 4 hours and restoration for 24 hours. (4)O+miR-451a mimic: The cells were co-cultured with miR-451a mimic for 24 hours, followed by OGD/R. (5)O+ NSC23766: The cells were treated with a Rac1 inhibitor, NSC23766, for 24 hours, followed by OGD/R. (6)O+miR-451a mimic + NSC23766: The cells were co-cultured with miR-451a mimic and NSC23766 for 24 hours, followed by OGD/R. (7)S + NSC23766: The cells were treated by NSC23766 for 24 hours.

**1.4 Information of primers for qRT-PCR of miRNAs**

| **miRNA names** | **Primer sequences** |
| --- | --- |
| mmu-miR-451a-F | 5' GGG GAA ACC GTT ACC ATT A 3' |
| mmu-miR-451a-R | 5' TGC GTG TCG TGG AGT C 3' |
| mmu-miR-486-3p-F | 5' TTT CGG GGC AGC TCA 3' |
| mmu-miR-486-3p-R | 5' CAG TGC GTG TCG TGG A 3' |
| mmu-miR-106b-5p-F | 5' GGG GGT AAA GTG CTG AC 3' |
| mmu-miR-106b-5p-F | 5' GTG CGT GTC GTG GAG T 3' |
| mmu-miR-1931-F-R | 5' GTT ATG CAA GGG CTG GT 3' |
| mmu-miR-1931-R | 5' GTG CGT GTC GTG GAG T 3' |
| mmu-miR-7009-5p-F | 5' TTT GGG TTG GGG TCA G 3' |
| mmu-miR-7009-5p-R | 5' AGT GCG TGT CGT GGA GT 3' |
| mmu-miR-7069-5p-F | 5' AAA ATT GGG GGC CTG 3' |
| mmu-miR-7069-5p-R | 5' GTG CGT GTC GTG GAG T 3' |
| mmu-miR-3090-5p-F | 5' GGT TTT GTC TGG GTG GG 3' |
| mmu-miR-3090-5p-R | 5' AGT GCG TGT CGT GGA GT 3' |
| snoRNA202-F | 5' GGG GCT GTA CTG ACT TGA T 3' |
| snoRNA202-R | 5' AGT GCG TGT CGT GGA GT 3' |

**1.5 Information of primers for qRT-PCR of mRNAs**

| **mRNA names** | **Primer sequences** |
| --- | --- |
| GAPDH-F | 5' AGG TCG GTG TGA ACG GAT TTG 3' |
| GAPDH-R | 5' TGT AGA CCA TGT AGT TGA GGT CA 3' |
| IL-1b-F | 5' GAA ATG CCA CCT TTT GAC AGT G 3' |
| IL-1b-R | 5' TGG ATG CTC TCA TCA GGA CAG 3' |
| IL-6 -F | 5' AGC CAG AGT CCT TCA GAG AGA 3' |
| IL-6 -R | 5' GCC ACT CCT TCT GTG ACT CC 3' |
| TNF-a-F | 5' GAA CTG GCA GAA GAG GCA CT 3' |
| TNF-a-R | 5' CGA TCA CCC CGA AGT TCA GT 3' |
| Rac1-F | 5' ATG ACA GAT TGC GTC CCC TC 3' |
| Rac1-R | 5' TCG GCA ATC GGC TTG TCT TT 3' |

**1.6 Databases for target screening**

| **Database names** | **URL** |
| --- | --- |
| miRanda | http://www.miRNA.org/miRNA/home.do |
| miRDB | http://mirdb.org/ |
| miRTarBase | http://mirtarbase.cuhk.edu.cn/php/index.php |
| starBaseV3 | http://starbase.sysu.edu.cn/index.php |
| TargetScan7.2 | http://www.targetscan.org/vert_72/ |

**2. Supplementary results**


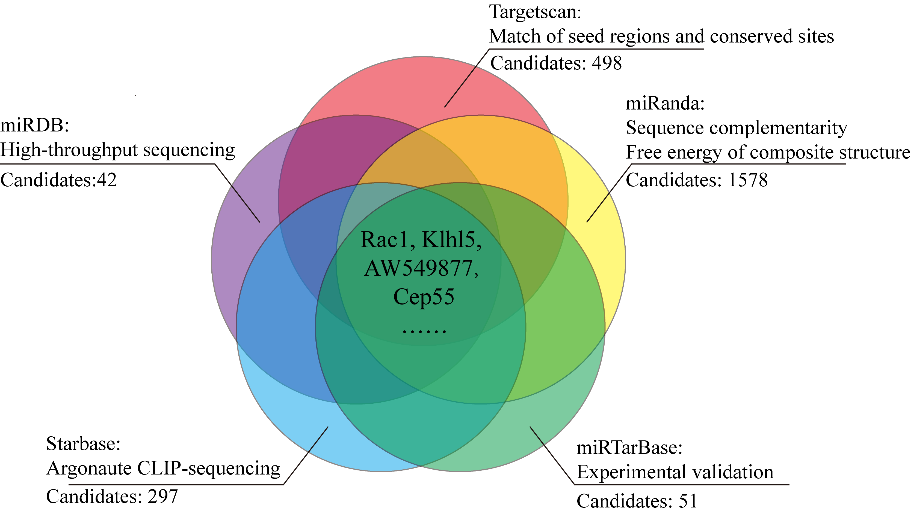
**2.1 Results of database searching for miRNA targets**

Five databases were searched for target prediction of miR-451a. Five candidates appeared five times in these databases.

**2.2 The statistical analysis of Western Blots**


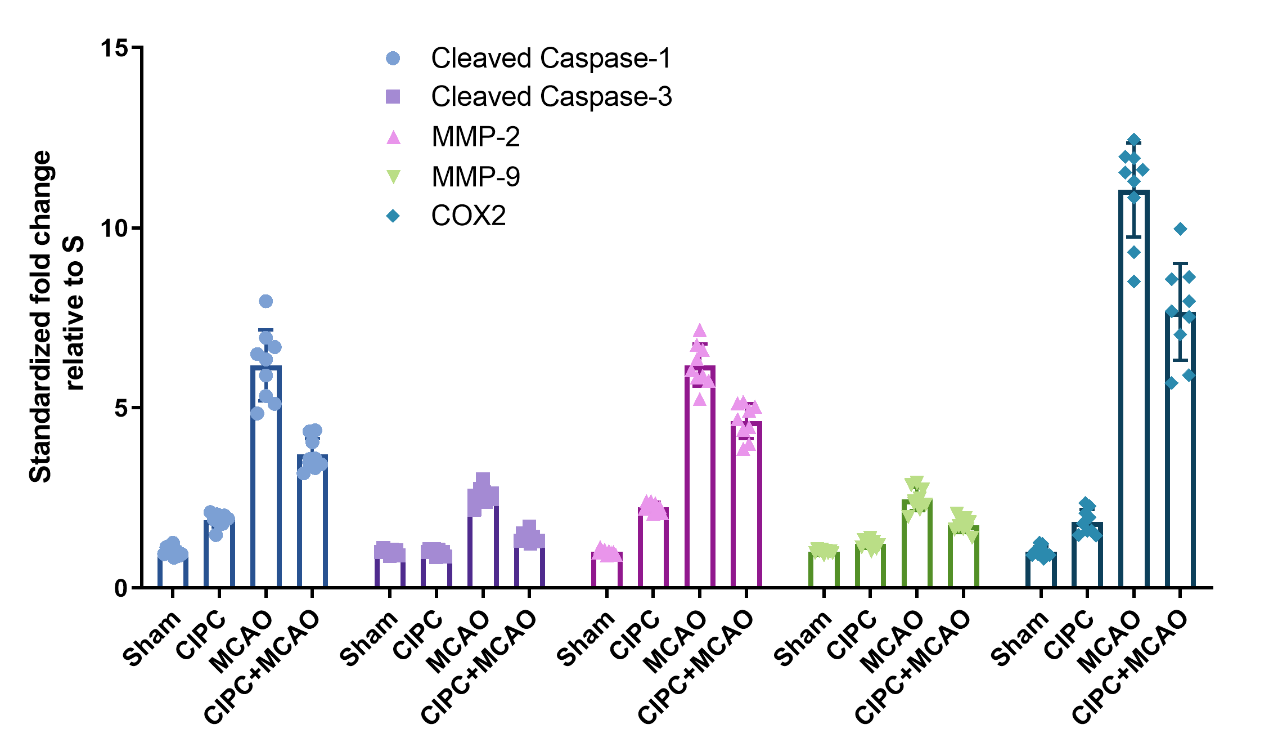


#

*#

*#

*

*

*#

*#

*

*#

*#

*

*#

*#

*#

*

**Figure S1**

The results of statistical analysis of Western Blots in Figure 1E. (* and # p < 0.05 compared to Sham group and MCAO group respectively) (CIPC: cerebral-IPC; MCAO: middle cerebral artery occlusion)


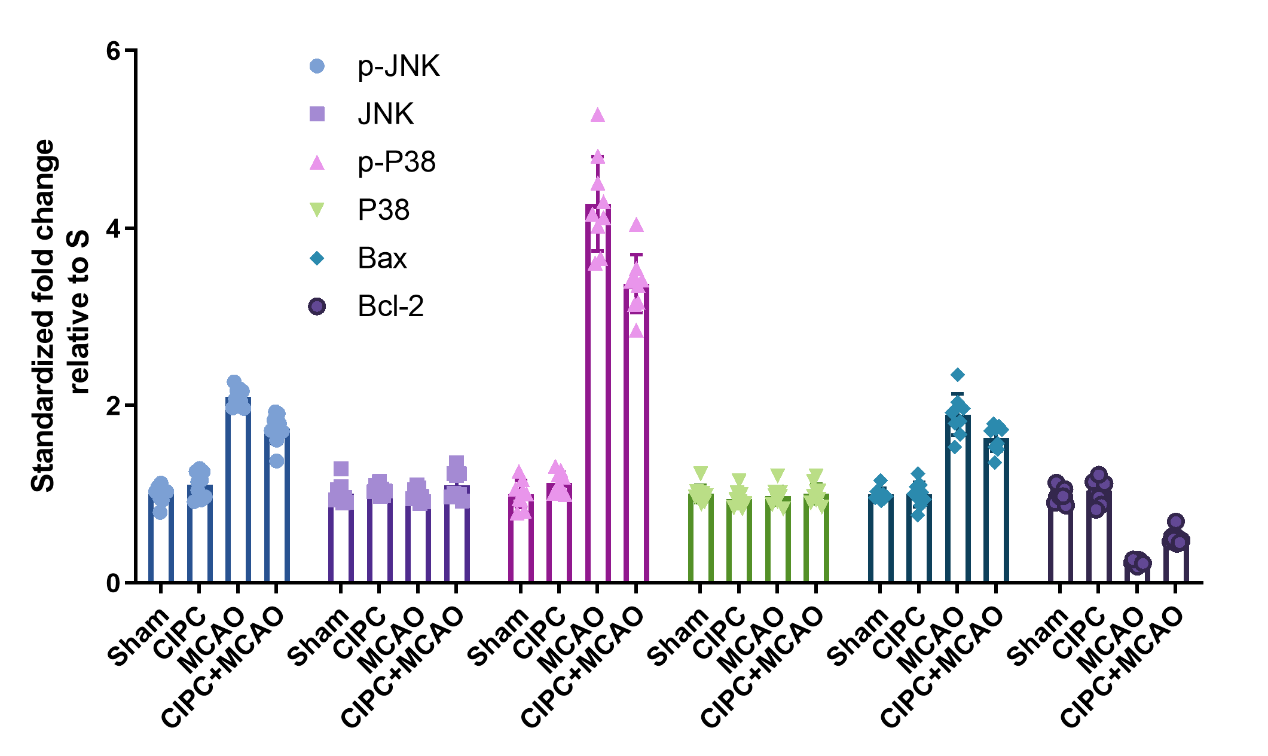


*

*#

#

#

#

#

*

*#

*#

*

*#

*

**Figure S2**

The results of statistical analysis of Western Blots in Figure 1F. (* and # p < 0.05 compared to Sham group and MCAO group respectively) (CIPC: cerebral-IPC; MCAO: middle cerebral artery occlusion)


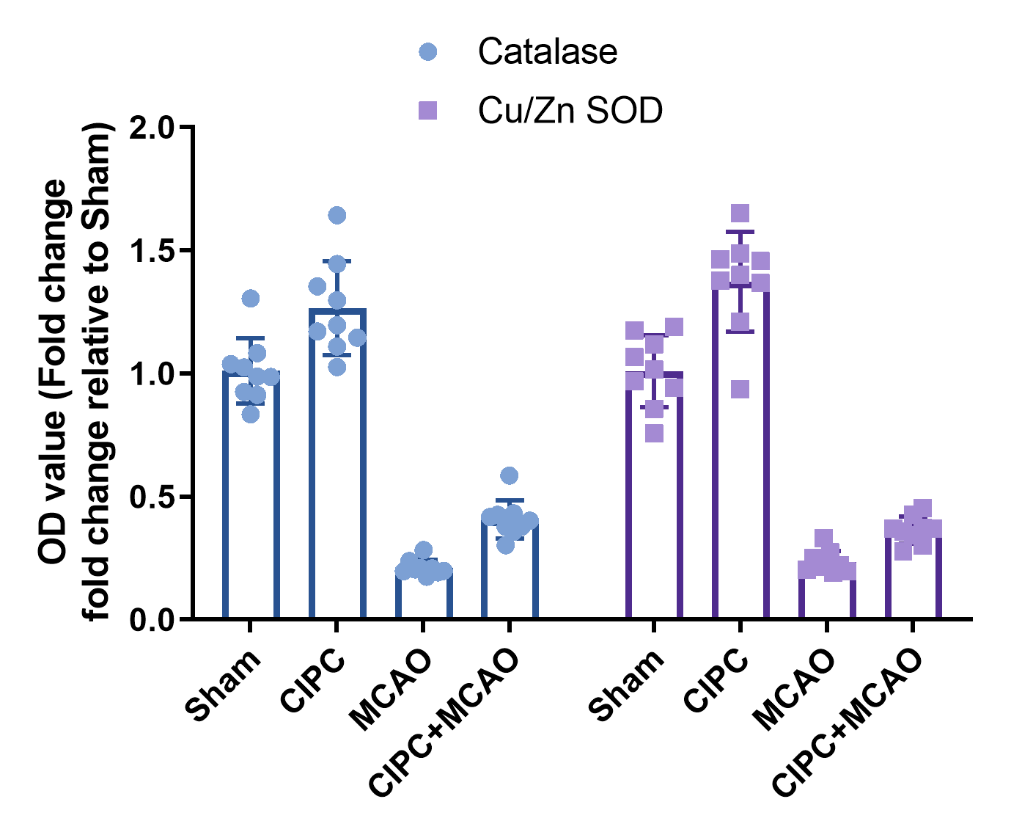


*#

*#

*

*#

*

*#

**Figure S3**

The results of statistical analysis of Western Blots in Figure 2E. (* and # p < 0.05 compared to Sham group and MCAO group respectively) (CIPC: cerebral-IPC; MCAO: middle cerebral artery occlusion)
